# Supplementary material for: Increased Angiogenesis and Lymphangiogenesis in Adenomyosis Visualized by Multiplex Immunohistochemistry
Source: Int J Mol Sci. 2022 Jul 29;23(15):8434. doi: 10.3390/ijms23158434 (PMC9369277; doi:10.3390/ijms23158434)
Supplement: Supplementary file 1 [file ijms-23-08434-s001.zip › Supplementary Table S1 - Multiplex staining panel.pdf]

Table S1. Multiplex staining panel.									
**Structure of interest**	**Antibody**	**Vectra Opal**	**Dilution**	**Incubation time**	**Company**	**Ref.**	**Clone**	**Clonality**	**Host**
Myometrium and vascular smooth muscle cells	α-SMA	Opal570	1:1500	30 min - RT	Dako	MO851	1A4	Monoclonal	Mouse
Angiogenesis marker	VEGF	Opal520	1:500	30 min – RT	Santa Cruz	Sc-152	A-20	Polyclonal	Rabbit
Blood vessels	CD31	Opal650	1:50	60 min – 37 °C	Dako	MO823	JC70A	Monoclonal	Mouse
Lymph vessels	Podoplanin (D2-40 antibody)	Opal540	1:50	30 min – RT	Bio Rad	MCA254 3	D2-40	Monoclonal	Human
Endometrium	CD10	Opal690	1:50	30 min - RT	monosan Sanbio	MONX1 0354	56C6	Monoclonal	Mouse
