# Supplementary figures and images for: Increased Angiogenesis and Lymphangiogenesis in Adenomyosis Visualized by Multiplex Immunohistochemistry
Source: Int J Mol Sci. 2022 Jul 29;23(15):8434. doi: 10.3390/ijms23158434 (PMC9369277; doi:10.3390/ijms23158434)

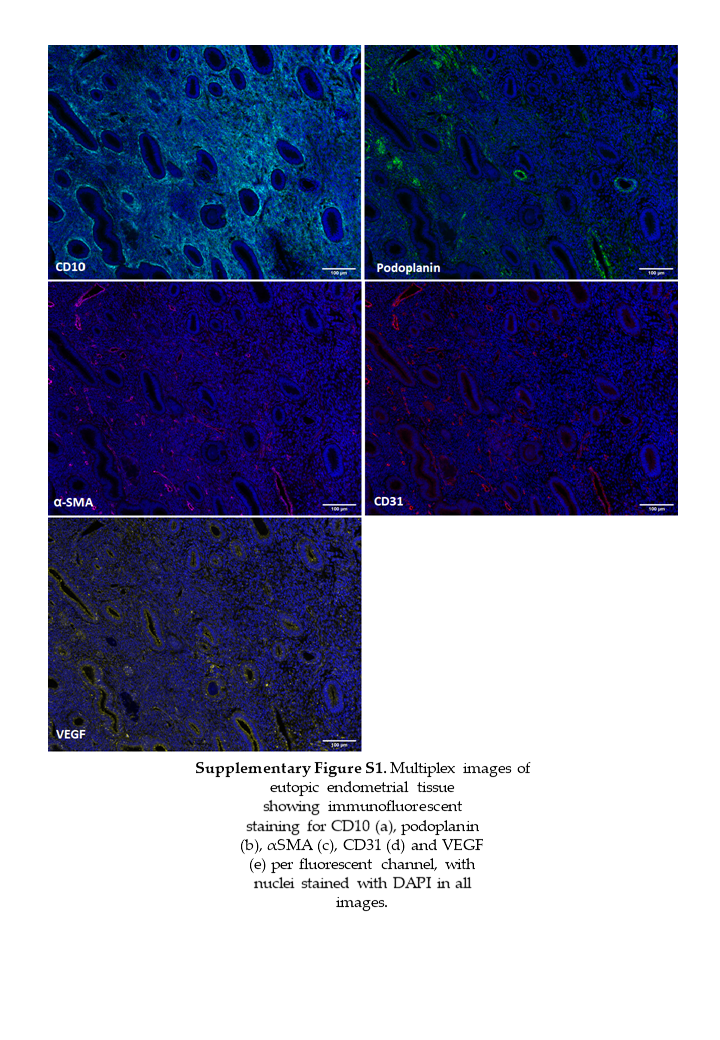

Supplement: Supplementary file 1 [file ijms-23-08434-s001.zip › Supplementary Figure S1.png]

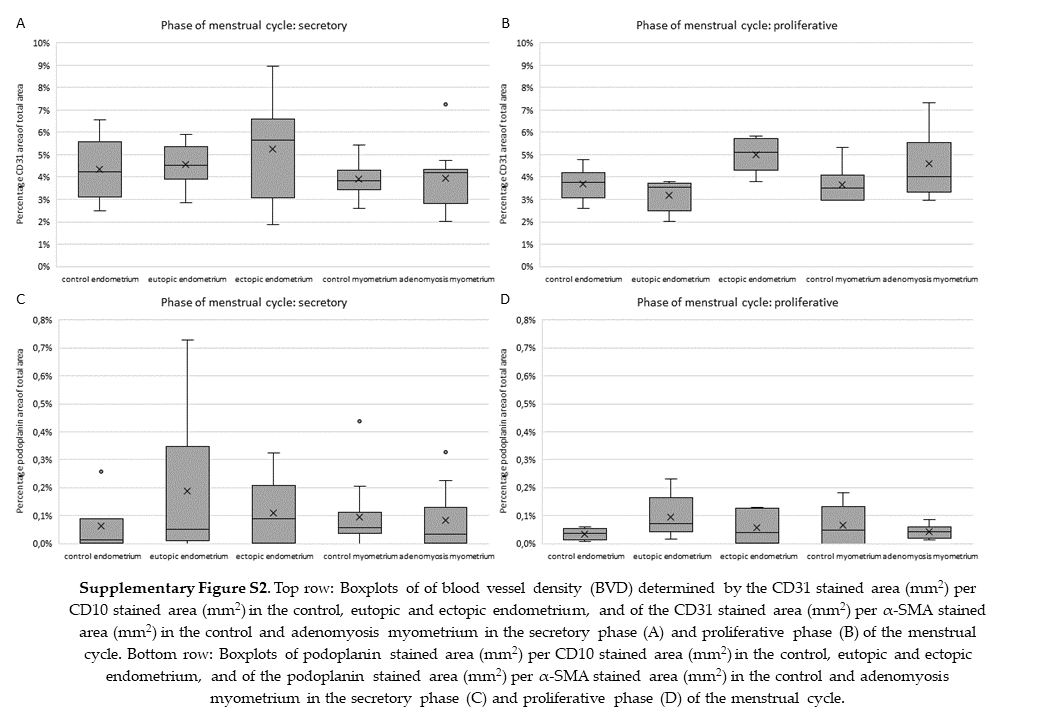

Supplement: Supplementary file 1 [file ijms-23-08434-s001.zip › Supplementary Figure S2.png]
